# Left main bronchial rupture: bronchoscopy and chest CT

**DOI:** 10.36416/1806-3756/e20240323

**Published:** 2024-12-17

**Authors:** Andrea Albuja Hidalgo, Nicolás Almeida-Arostegui, María Soledad Alonso Viteri

**Affiliations:** 1. Servicio de Neumología, Hospital Universitario de Torrejón, Madrid, España.; 2. Servicio de Radiología, Hospital Universitario Nuestra Señora del Rosario, Madrid, España.

**Figure 1:**
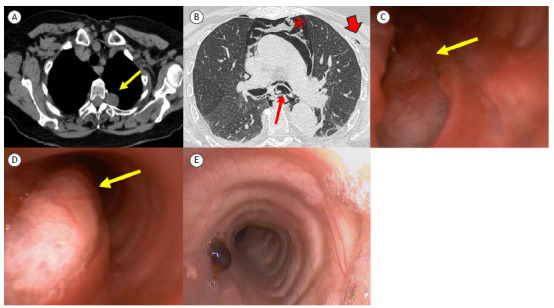
In A, axial unenhanced chest CT scan showing a left apical extrapleural lesion (arrow) arising from the intervertebral foramen, suggestive of a peripheral nerve sheath tumor (schwannoma). The diagnosis was confirmed after resection. In B, postoperative axial chest CT scan with lung window settings, showing a laceration of the posterior wall of the main bronchus (thin arrow). Note pneumomediastinum (asterisk) and mild subcutaneous emphysema (thick arrow). In C and D, bronchoscopic images showing the laceration during inhalation (arrow in C) and the lung protruding into the bronchial lumen during exhalation (arrow in D). In E, a bronchoscopy performed after four months of conservative treatment shows a small amount of granulation tissue on the posterior wall of the left main bronchus, with almost complete closure of the laceration.

A 72-year-old woman presented to our clinic with chronic cough. A chest CT scan showed an extrapleural lesion in the left upper lobe, suggestive of intercostal nerve schwannoma (Figure 1A). The patient was admitted for tumor resection by left video-assisted thoracoscopy. Intubation with a left-sided double-lumen endobronchial tube was required. The patient was admitted to the ICU and was successfully extubated. Twenty-four hours later she presented with subcutaneous emphysema in the neck, and a chest CT scan showed pneumomediastinum (Figure 1B). An emergency bronchoscopy was performed, showing a 3-cm rupture of the left main bronchus (Figures 1C and 1D). Given the clinical stability of the patient, muscle relaxants, oxygen therapy, and cough suppressants were prescribed, and a decision was made for conservative management with close monitoring. Four months later, a bronchoscopy was performed, showing scar tissue and resolution of the rupture (Figure 1E). Bronchial rupture after intubation with a double-lumen endobronchial tube has rarely been reported,^1^ and mortality can be as high as 23%. Although bronchoscopy is the gold standard, chest CT can aid in locating the injury.^2^ Mild symptoms and small ruptures can be managed with conservative treatment.^1^
